# Microbial community and functions associated with digestion of algal polysaccharides in the visceral tract of *Haliotis discus hannai*: Insights from metagenome and metatranscriptome analysis

**DOI:** 10.1371/journal.pone.0205594

**Published:** 2018-10-11

**Authors:** Bo-Hye Nam, Jisung Jang, Kelsey Caetano-Anolles, Young-Ok Kim, Jung Youn Park, Hawsun Sohn, Sook Hee Yoon, Heebal Kim, Woori Kwak

**Affiliations:** 1 Biotechnology Research Division, National Institute of Fisheries Science, Busan, Republic of Korea; 2 Interdisciplinary Program in Bioinformatics, Seoul National University, Seoul, Republic of Korea; 3 C&K Genomics, Seoul, Republic of Korea; 4 Department of Agricultural Biotechnology and Research Institute of Agriculture and Life Sciences, Seoul National University, Seoul, Republic of Korea; 5 Cetacean Research Institute, National Institute of Fisheries Science, Nam-gu, Ulsan, Republic of Korea; Korea University, REPUBLIC OF KOREA

## Abstract

*Haliotis discus hannai*, a species of Pacific abalone, is a highly valuable food source throughout Northeast Asia. As *H*. *discus hannai* primarily feed on brown algae and largely extract their energy from algal polysaccharides, understanding the mechanisms by which they digest algal polysaccharides is essential for elucidating their energy metabolism. Gut microbes, as well as the host animal, are involved in the process of polysaccharide degradation. To identify algal polysaccharide-digestion mechanisms and their origin, we analyzed the metagenome and metatranscriptome of abalone visceral extracts. Microbial communities were characterized using the 16S rRNA gene sequences in the metagenome and our results differed significantly from those of previous studies using traditional microbiological methods such as bacterial cultivation and cloning. A greater diversity of bacterial taxa was identified here than was previously identified using cultivation methods. Furthermore, the most abundant bacterial taxa also differed from previous studies, which is not common when comparing the results of bacterial culturing with those of molecular methodologies. Based on the metatranscriptome, overall functions were identified and additional analyses were performed on the coding sequences of algal polysaccharide-digestive enzymes, including alginate lyase. Results of the transcriptomic analyses suggest that alginate lyase in the visceral extracts of *H*. *discus hannai* was produced by the host itself, not by visceral bacteria. This is the first next-generation sequencing study performed on abalone to characterize the visceral microbiota and the source of the ability to digest algal polysaccharides by analyzing the metagenome and metatranscriptome together.

## Introduction

Abalone are a group of marine snails which constitute a single genus, *Haliotis* in the *Haliotidae* family [1]. Within the genus *Haliotis*, 57 abalone species are classified principally based on their habitat [2]. One characteristic of abalone is that they consume only macroalgae, primarily red and brown algae [3]. The main food of *Haliotis discus hannai* is *Laminaria japonica*, a species of brown algae [3]. As polysaccharides are used for energy storage in brown algae, it is crucial to understand the polysaccharide digestion mechanism of abalone to understand their energy metabolism. Brown algae are composed largely of polysaccharides such as alginates, *β*-glucans (laminarans), cellulose, heteroglycans, and fucoidans [4, 5]. In particular, alginates constitute up to 40% of the dry mass of brown algae [6]. Therefore, digestive enzymes such as alginate lyase, *β*-1,3-glucanase, cellulase, and agarase are essential for abalone to obtain nutrients from these algal polysaccharides. The ability of abalone visceral extracts to digest algal polysaccharides has been investigated previously using culturing methods on agar plates. For example, 70% of bacteria isolated from the gut of *Haliotis gigantea* showed an ability to degrade alginate on agar plates [7]. In *H*. *discus hannai*, *Vibrio halioticoli*, a species of gut microbe, produced acetic acid and volatile short chain fatty acids on a growth medium that included sodium alginate due to alginate degradation by multiple enzymes [8]. Likewise, the gut bacteria of abalone can help it obtain energy by supporting the degradation of algal polysaccharides. Gut bacterial enzymes degrade polysaccharides to smaller components that are more easily absorbed by their host [9]. Bacterial culturing methodologies were useful for identifying several species and evaluating their ability to digest algal polysaccharides. However, the results of culturing methods have not accurately reflected the microbial community due to the differing oxygen conditions of the experimental environment and the anaerobic conditions within the actual gut [10]. Additionally, the artificial high-nutrient content found in the growth media used for these cultivation methods differs significantly from the conditions within the visceral environment, which can greatly affect both the relative abundance of bacteria and the dominant species present [10]. To overcome the limitations of culturing methods, we applied high-throughput sequencing to visceral extracts of *H*. *discus hannai*. This is the first next-generation sequencing (NGS) study to use 16S rRNA gene sequences to characterize the abalone visceral microbiota. By analyzing the abalone visceral metagenome, we characterized the bacterial community and bacterial abundance in detail.

Until now, both the composition and function of the bacterial community within abalone viscera were poorly understood. In this study, we examined the visceral metatranscriptome of *H*. *discus hannai* to determine the functional profile of transcripts from the visceral environment. Additionally, we focused on transcripts related to the energy-yielding pathways that allow *H*. *discus hannai* to digest brown algae. By analyzing the phylogeny of related enzymes to identify their origin, we determined the roles of *H*. *discus hannai* and its gut bacteria in extracting energy from the indigestible polysaccharides of brown algae.

## Materials and methods

### Sample collection

*Haliotis discus hannai* (8-cm shell length) samples used for metagenomic and metatranscriptomic analyses of the abalone digestive system were purchased from Wan Island, Korea in February of 2016. Only the alimentary canal, extracted through viscera dissection, was used in the analysis.

### Library construction for bacterial community analysis

The visceral extracts of all samples from alimentary canal were released and pooled into Eppendorf tubes. The mixture of visceral extracts was homogenized with sterile plastic pestles. Genomic DNA was isolated from intestine of abalone using PowerSoil DNA Isoloation kit (Mo Bio). To amplify the variable V3 and V4 region of the 16S rRNA gene, we performed PCR as follows: 10 ng of DNA and 2× Quick Taq HS DyeMix Z (Toyobo) were used to make a 30-μl reaction volume to perform PCR with the following PCR conditions: 95°C for 3 min; 25 cycles of 95°C for 30 s, 55°C for 30 s, and 72°C for 30 s; followed by a final extension of 72°C for 5 min and a hold at 4°C. The primers used in this analysis were as follows:

1st PCR Forward Primer:

5' -TCGTCGGCAGCGTCAGATGTGTATAAGAGACAGCCTACGGGNGGCWGCAG-3'

1st PCR Reverse Primer:

5'-GTCTCGTGGGCTCGGAGATGTGTATAAGAGACAGGACTACHVGGGTATCTAATCC-3'

The PCR products were sequenced from an Illumina MiSeq 300-bp paired-end library, using the standard protocol. Descriptions of the data are provided in S1 Table.

### Metatranscriptome library construction

The whole transcriptome sequencing (WTS) library was constructed with the TruSeq Stranded Total RNA Sample Prep Kit (Illumina). Ribosomal RNAs were excluded from the total RNA using an rRNA removal kit, followed by RNA purification with RNAClean XP beads (Beckman Coulter). The remaining RNA was fragmented with EFP Mix under PCR conditions of 94°C for 6 min and a 4°C hold. The fragmented RNAs were used to synthesize first strand cDNA using reverse transcriptase and random primers under PCR conditions of 25°C for 10 min, 42°C for 15 min, 70°C for 15 min, and a 4°C hold. For double stranded cDNA synthesis from the first strand cDNAs, Second Strand Marking Master Mix was used under PCR conditions of 16°C for 1 h and a 4°C hold. Library construction was performed using the Illumina NextSeq 500 reagents following the manufacturer’s protocol. Details are provided in S2 Table.

### Metagenomic analysis of the bacterial community

The quality of generated reads was checked using FASTQC software [11] and adapter sequences were removed using Trimmomatic software [12]. Paired-end reads were merged using FLASH software [13] to generate a single read including the V3-V4 region of the 16S rRNA gene. Finally, community analysis was conducted using MG-RAST software [14] against the Greengenes database [15].

### Metatranscriptome analysis

Quality control processing was conducted using FASTQC and Trimmomatic. The Trinity *de novo* transcriptome assembly tool was used to identify genes expressed in the visceral extract of abalone [16]. Summary statistics for the total transcriptome obtained from Trinity are shown in S3 Table. Using paired-end read remapping, we filtered out misassembled candidate transcripts displaying low paired-end read mapping from the metatranscript assembly. Candidate coding regions of the assembled transcripts were identified using Transdecoder software [17]. Functional annotation and the origins of coding region sequences were identified by cross-referencing the dataset against the nt database (non-redundant nucleotide database) using BLASTn (e-value < 1e–5, > 50% sequence similarity, and > 50% alignment coverage). Additionally, the overall functions of the metatranscriptome were profiled using the SAMSA2 pipeline [18]. At the preprocessing step, PEAR software was used to merge paired-end reads, and low-quality sequences and adaptor contamination of the merged reads was removed using Trimmomatic [19, 20]. Among the cleaned reads, ribosomal reads were removed using the SortMeRNA program with the SILVA and Rfam rRNA databases [21–23]. Next, without read assembly, a BLAST-like algorithm in the DIAMOND program was applied to annotate the remaining reads using two databases, NCBI’s RefSeq microbial genomes database and the SEED subsystems hierarchical database [24–26]. Finally, annotation results were visualized using scripts included in the SAMSA2 package [18].

### Phylogenetic tree construction

Publicly available alginate lyase sequences were collected and used to construct a phylogenetic tree including three sequences obtained from this study. The publicly available sequences included complete bacterial, marine gastropoda, and algal alginate lyase coding sequences. The PRANK program was used for multiple sequence alignment of the dataset [27], and poorly aligned positions were examined and eliminated using the Gblocks program [28]. Sequence identity between aligned sequences was calculated using BioEdit software and some identical sequences were excluded [29]. Phylogenetic trees for the alginate lyase coding nucleotide sequences were constructed using the neighbor joining (NJ) method after replication of 1000 bootstraps using MEGA7 software [30–32]. Maximum composite likelihood was selected as a substitution model and substitution rates among all sites were assumed to be uniform [33].

## Results and discussion

### Microbial community of abalone visceral extract

**Fig 1:**
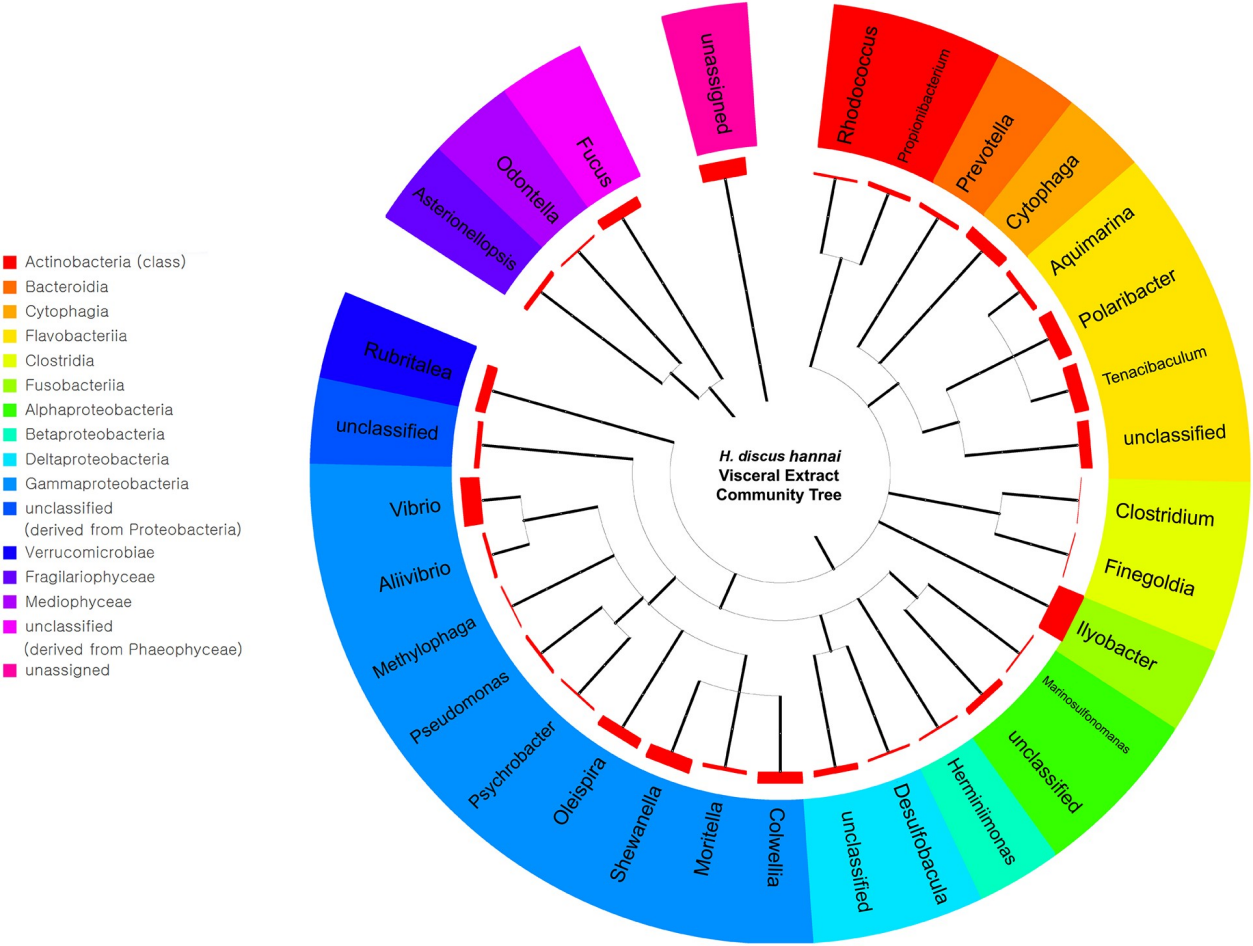
Microbial community in abalone visceral extract. Community tree of abalone visceral extract based on 16S rRNA gene sequences at the genus level. The tree displays the evolutionary relationships between bacterial genera. The thickness of the red bar indicates the relative abundance of each genus.

We identified and quantified the abalone visceral microbiota at each taxonomic level from the visceral metagenome. Summary statistics of the metagenome for merged paired-end reads are provided in S3 Table. Alpha-diversity was 9.812 and a rarefraction curve is shown in S1 Fig. The bacterial taxa identified within the community and their relative abundance in the viscera of abalone from phylum to genus levels. At the genus level, the evolutionary relationships of the bacteria from the community were visualized along with their relative abundances (Fig 1). A greater diversity of taxa was identified here than in previous studies, which relied on more traditional microbiological methods such as bacterial culturing and cloning. While the total number of bacterial genera identified was less than ten in cultivation studies, paired-end reads from the metagenome in this study were classified into 43 genera [9, 34]. Additionally, differences in the dominant taxa and their relative abundances between previous cultivation studies and the present NGS study were identified at all taxonomic levels. From phylum to genus level, the bacterial abundance at each level in this study was consistent in that the proportion of the two dominant taxa constituted 41% and 33% of the microbiota, respectively. Among microbes identified at the phylum level, *Tenericutes* and *Fusobacteria* were the dominant microbes, which constituted 41.4% and 33.1% of the total microbial community, respectively. At the genus level, the majority of *Tenericutes* (phylum) was *Mycoplasma* (41.3%) and the majority of *Fusobacteria* (phylum) was *Ilyobacter* (33.0%). This suggests low diversity within each of these dominant phyla.

Most abalone gut microbes, including *Mycoplasma* and *Ilyobacter*, have been found to degrade agar when cultured on agar plates [7, 35]. As agar is a mixture of polysaccharides derived from the cell walls of algae, agar degradation is indirect evidence of the ability to digest algal polysaccharides [36]. The agar-degrading bacteria can help the abalone to digest algae within its visceral tract. In the case of *H*. *discus hannai*, the digestive enzymes of gut microbes help the host utilize algae containing large amounts of polysaccharides [37].

In addition to *Mycoplasma*, *Vibrio*, which also demonstrated agar-degrading ability in culture-dependent studies, has previously been identified as a dominant genus in the abalone gut [7, 38]. However, *Vibrio* accounted for only 1.63% of the community at the genus level in our results. These conflicting results can be accounted for by temperature and differences in methodologies. First, the room temperature used to culture bacterial isolates in previous studies promoted the growth of *Vibrio* from abalone gut [39]. In contrast to those cultivation temperatures, our samples were collected at cold winter temperatures. The daily average air temperature of Wan Island ranged from 11.8°C to 14.3°C and the water temperature near Wan Island ranged from 4.3°C to 10.2°C (Feb., 2016, Korea Meteorological Administration) [40, 41]. In addition to temperature, culture media and aerobic conditions were potential causes of overestimation of the proportion of *Vibrio*. Most of the culture-based studies used alginate-peptone-yeast extract (APY) as a culture medium under aerobic conditions following the method described by Sawabe et al. [42]. This artificial medium and the aerobic conditions differ drastically from the natural conditions within the abalone visceral tract. As a result, *Vibrio* showed greater abundance in the culture-based study than in the molecular data of this study.

Another novel discovery was the high abundance of the *Fusobacteria* phylum, including the *Ilyobacter* genus (also known as *Lysobacter*). Previous studies of the gut microbiota from abalone, including *H*. *discus hannai*, identified *Fusobacteria*, but its proportion was not dominant [35, 38] as found in our results. This difference was likely due to the obligatory anaerobic and asaccharolytic nature of *Fusobacteria* and *Ilyobacter*. In most of the preparation methods used for the gut microbiota it is difficult to maintain the obligatory anaerobic conditions. As a result, the growth of obligate anaerobes, including *Fusobacteria*, is inhibited [43]. Additionally, the asaccharolytic nature of *Fusobacteria* inhibits its ability to produce energy under aerobic conditions [44]. Because of these two characteristics related to oxygen, the abundance of *Fusobacteria* and *Ilyobacter* would be underestimated when using traditional methods as opposed to a metagenomic approach.

In the first NGS analysis of abalone visceral extract described here, more diverse taxa were identified in detail, which were beyond the detection range of traditional methods, such as bacterial culturing and cloning. Moreover, the relative abundances of microbes could be precisely quantified based on read counts while traditional methods are dependent on ambiguous indicators such as colony forming units (CFU). These two improvements helped us to survey the actual state of the microbial community in abalone viscera.

### Functional profile of the abalone visceral metatranscriptome

**Fig 2:**
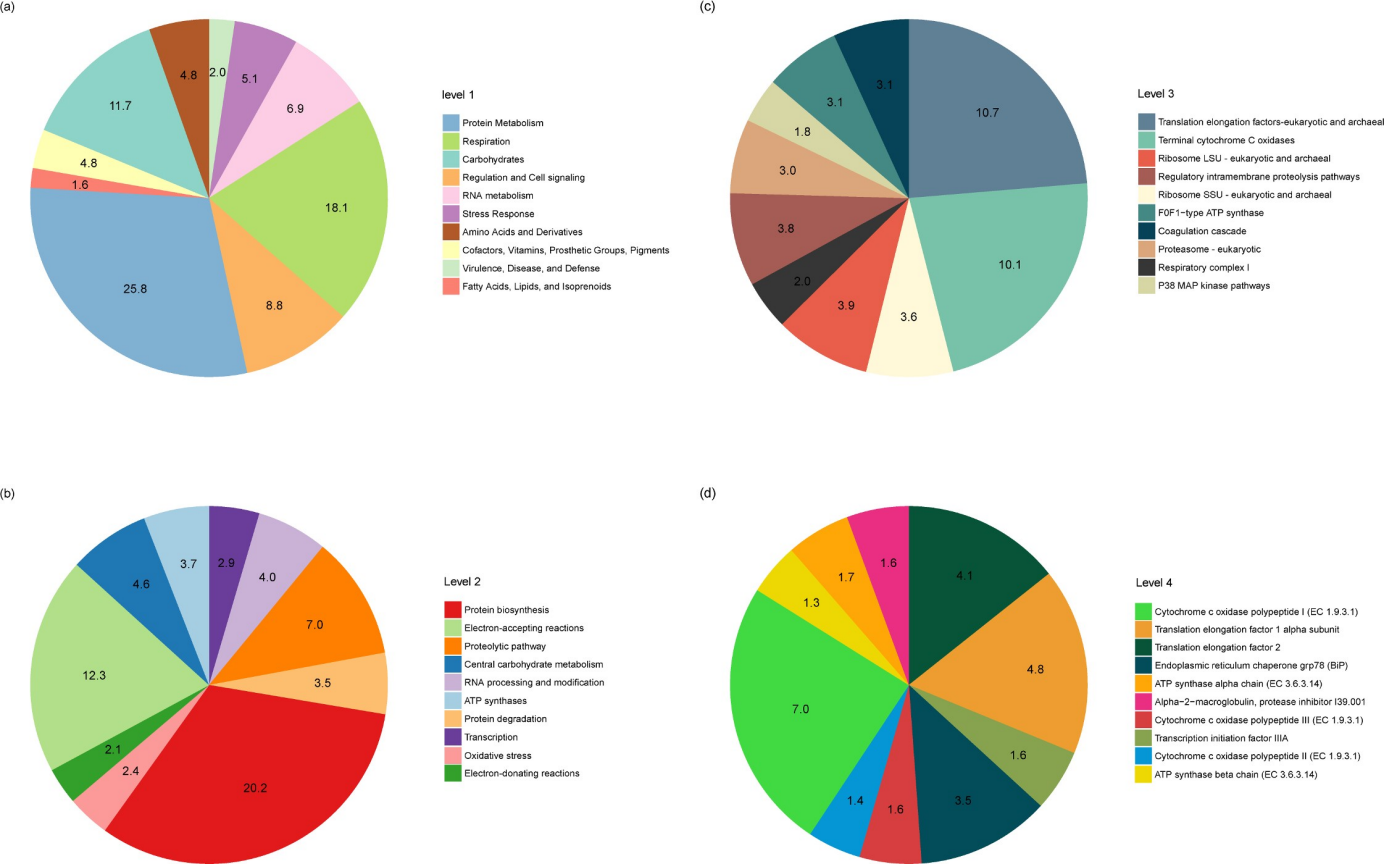
Hierarchical profiling of functional activities based on the SEED subsystems hierarchical database. Each pie chart shows the proportion of the top ten functions hierarchically at: (a) level 1, (b) level 2, (c) level 3, and (d) level 4.

We annotated metatranscriptome functions using two databases: the SEED subsystems and RefSeq microbial genome databases. Fig 2 shows the annotation results from the SEED subsystems hierarchical database. At the most comprehensive hierarchy level, the ten most abundant major functions accounted for 87.8% of all annotated functions. The most abundant function present was protein metabolism (25.8%), followed by respiration (18.1%), carbohydrates (11.7%), regulation and cell signaling (8.8%), RNA metabolism (6.9%), and stress response (5.1%). A large proportion of the protein metabolism category corresponded to protein biosynthesis and protein degradation. Respiration, the second most predominant function, consisted of four subsystems and three of them (electron-accepting reactions, electron-donating reactions, and ATP synthases) were also prevalent in level 2. The carbohydrates term included 11 carbohydrate metabolisms and the representative function was central carbohydrate metabolism. The regulation and cell signaling group contained four subsystems, including a proteolytic pathway. RNA metabolism consisted of transcription, RNA processing, and RNA modification. The subsystems of stress response were classified based on causes of stress, such as oxygen and heat shock. Likewise, major subsystems of predominant functions at higher levels were also predominant at the lower level. For example, in level 3, ten of the predominant subsystems were included in three categories of level 1 such as ‘protein metabolism’, ‘respiration’, and ‘regulation and cell signaling’. In level 4, the total number of functions was 1957 and the top ten predominant functions accounted for 28.6% of the total.

The annotation results from the NCBI RefSeq microbial genomes database are summarized in Table 1. The top ten most abundant functions were similar to the results from the hierarchical level 4 of the SEED database. For example, cytochrome and translation elongation factors were predominant in both sets of annotation results. Furthermore, functions related to respiration, carbohydrate metabolism, and protein metabolism were also predominant. As these three functional categories play fundamental roles in all cells in the visceral environment, their abundances were higher than those of other functions. For example, protein synthesis and degradation, as well as respiration and central carbohydrate metabolism, are essential for all living organisms. Given that this is the first metatranscriptomic study of visceral extracts from marine mollusks, we compared our results to previous studies looking at transcriptomic data from host tissues in general. In comparison with the digestive gland of *H*. *discus discus*, transcripts related to cytochrome c oxidase and elongation factors were abundant in the visceral metatranscriptome. Alternatively, transcripts encoding digestive enzymes, such as cellulase and endo-1,4-β-glucanase (laminarase), were more abundant in *H*. *discus discus* because the transcriptome was collected from digestive gland [45]. In the case of visceral tissue from a Korean land snail (*Koreanohadra kurodana*), nucleotide metabolism was less abundant and protein metabolism was more abundant in *H*. *discus hannai* [46]. In short, the overall functional abundance of visceral extract was generally similar to that of host tissue except for protein metabolism function. The reason for the difference in functional activity of protein metabolism can ascribed to the active protein metabolism of the visceral microbiota.

**Table 1:** The top ten enriched functional categories from the metatranscriptome obtained from the RefSeq microbial genomes database.

Functional annotation	Proportion (%)
actin, cytoplasmic 2	5.93
cytochrome c oxidase subunit I	3.90
translation elongation factor EF-1 subunit alpha	2.67
molecular chaperone DnaK	2.49
cytochrome b	2.09
aldehyde dehydrogenase family protein	2.01
elongation factor 1-alpha, partial	1.24
50S ribosomal protein L14	1.15
50S ribosomal protein L3, partial	1.06
molecular chaperone HtpG	1.05

### Brown algae polysaccharide-degrading enzymes represented in the visceral metatranscriptome

**Fig 3:**
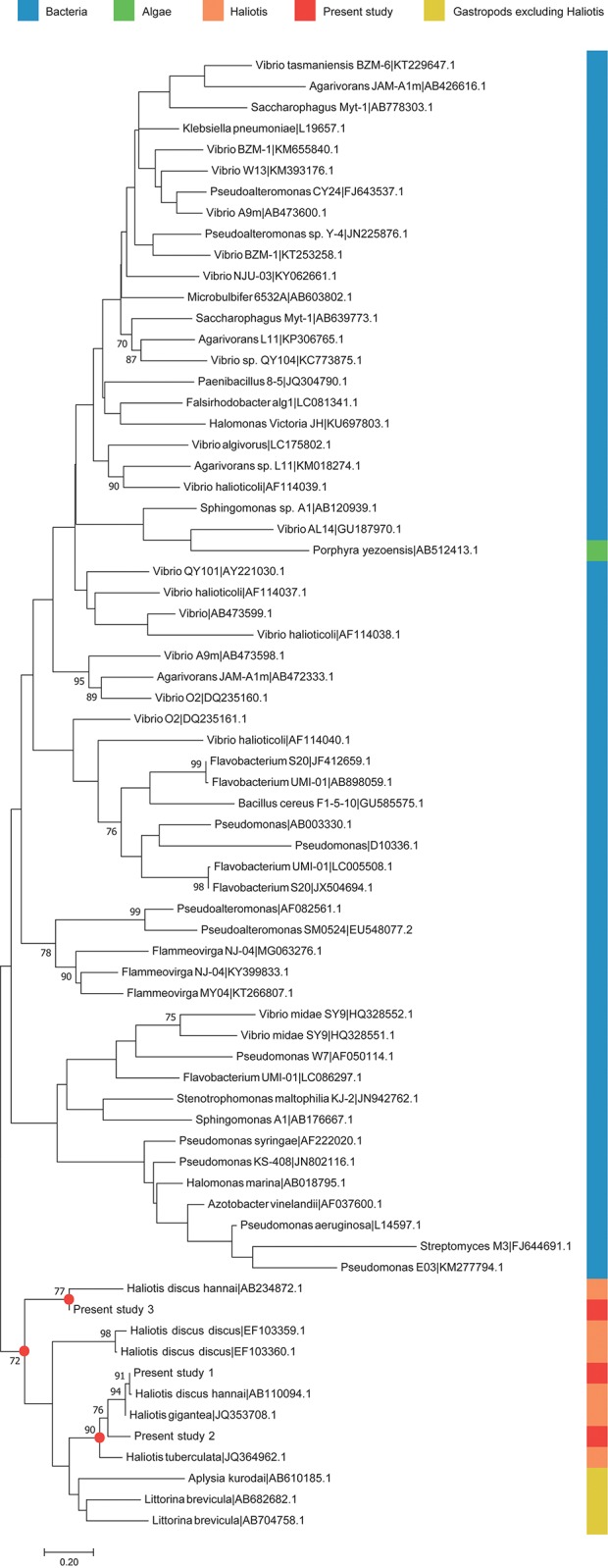
Phylogenetic tree of alginate lyase coding sequences created using the neighbor-joining method. Percentages of bootstrap values over 70% are displayed left of the nodes. Branch length equates to evolutionary distance. The origins of publicly available sequences are given with the corresponding GenBank accession numbers. The different origins of the publicly available sequences are distinguished by different colored boxes to the right of the terminal nodes. Nodes that include sequences from this study are highlighted with a red dot.

Through metatranscriptomic analysis, we identified various expressed genes and related functions. Among them, we focused on genes involved in the digestion of algal polysaccharides and host energy metabolism. The main food and energy source of *H*. *discus hannai* is brown algae, *Laminaria* in particular [3]. Algal polysaccharides, such as alginate, β-glucans (laminarans), cellulose, heteroglycan, and fucoidan are major components of the brown algae [4, 5]. In general, herbivores derive a large amount of energy from dietary polysaccharides and require support from gut their microbiota to supply the digestive enzymes needed to improve digestion [47]. In addition to microbial breakdown, some herbivorous mollusks, including abalone, secrete their own polysaccharide-degrading enzymes [48]. In the case of *H*. *discus hannai*, alginate lyase has previously been isolated from the hepatopancreas using chromatography and cDNA cloning [49, 50]. Additionally, several species of gut bacteria from *H*. *discus hannai* showed an ability to degrade alginate during culturing experiments on agar plates [8, 42]. Although alginate digestion abilities have been identified previously in both *H*. *discus hannai* and their associated gut bacteria, the digestion mechanism in the visceral tract had not yet been explored. We examined the presence and origin of algal polysaccharide-degrading enzymes within the visceral extract metatranscriptome for the first time. After open reading frame (ORF) prediction, we performed a BLAST search of all sequences from the metatranscriptome. Two of the major enzymes required for algal polysaccharide degradation, alginate lyase and laminarinase, were identified. BLAST results suggest that *H*. *discus hannai* is the source of both enzymes in the dataset (Table 2 & Table 3). We constructed a phylogenetic tree of the alginate lyase coding sequences to clarify the origin of the alginate lyase coding transcripts identified in this study (Fig 3). The sequences identified in this study grouped with other sequences from *Haliotis* (Fig 3). Sequences from marine gastropods grouped together and were distinguishable from the groups of bacterial and algal alginate lyases. Although the origin of the alginate lyase-encoding transcript was identified as *H*. *discus hannai*, it does not mean that the visceral bacteria did not have any role in degrading algal polysaccharides within the abalone gut. In general, the gut microbiota of aquatic invertebrates supports the energy metabolism of its host by catalyzing the degradation of large polymers into smaller molecules, such as short chain fatty acids [8, 51]. Even though the mechanism has not yet been explained at the molecular level, the ability to degrade algal polysaccharides was identified in many species of visceral bacteria isolated from abalone in culturing experiments [7–9]. Likewise, previous studies have revealed that genes from both abalone and their visceral microbiota play a role in algal digestion. For instance, alginate lyase is essential for degrading alginate, which is the major component of brown algae. The results from this study, including BLAST analysis, a phylogenetic tree, and microbial community assessments, suggest that alginate lyase in the viscera of *H*. *discus hannai* was produced by the host itself, not by the visceral bacteria.

**Table 2:** BLAST analysis results for alginate lyase coding transcripts using the non-redundant nucleotide database.

Description	Identity (%)	Length	Mismatch	E-value	Accession no.
***Haliotis discus hannai*** hdalex-1 mRNA for alginate lyase, complete cds	99.4	321	2	1.72E–162	AB234872.1
***Haliotis gigantea*** alginate lyase mRNA, complete cds	86.2	711	89	0	JQ353708.1
***Haliotis discus hannai*** HdAly mRNA for alginate lyase, complete cds	99.4	840	5	0	AB110094.1

**Table 3:** BLAST analysis results for laminarase-coding transcripts using the non-redundant nucleotide database.

Description	Identity (%)	Length	Mismatch	E-value	Accession no.
***Haliotis discus discus*** endo 1,4-beta D-glucanase 1 mRNA, complete cds	99.5	582	3	0	EF103350.1

## Supporting information

S1 Fig Rarefaction curve of annotated species richness in visceral extract of *Haliotis discus hannai*. (DOCX)

S1 Table Summary statistics of whole genome sequencing data generated for three Pacific abalone species. (DOCX)

S2 Table Summary statistics for total transcriptome assembly from visceral extract using Trinity. (DOCX)

S3 Table Summary statistics for 16S rRNA library data used in community analysis of visceral extract of *Haliotis discus hannai*. (DOCX)
