# Supplementary material for: Microbial community and functions associated with digestion of algal polysaccharides in the visceral tract of Haliotis discus hannai: Insights from metagenome and metatranscriptome analysis
Source: PLoS One. 2018 Oct 11;13(10):e0205594. doi: 10.1371/journal.pone.0205594 (PMC6181387; doi:10.1371/journal.pone.0205594)
Supplement: S1 Fig — (DOCX) [file pone.0205594.s001.docx]

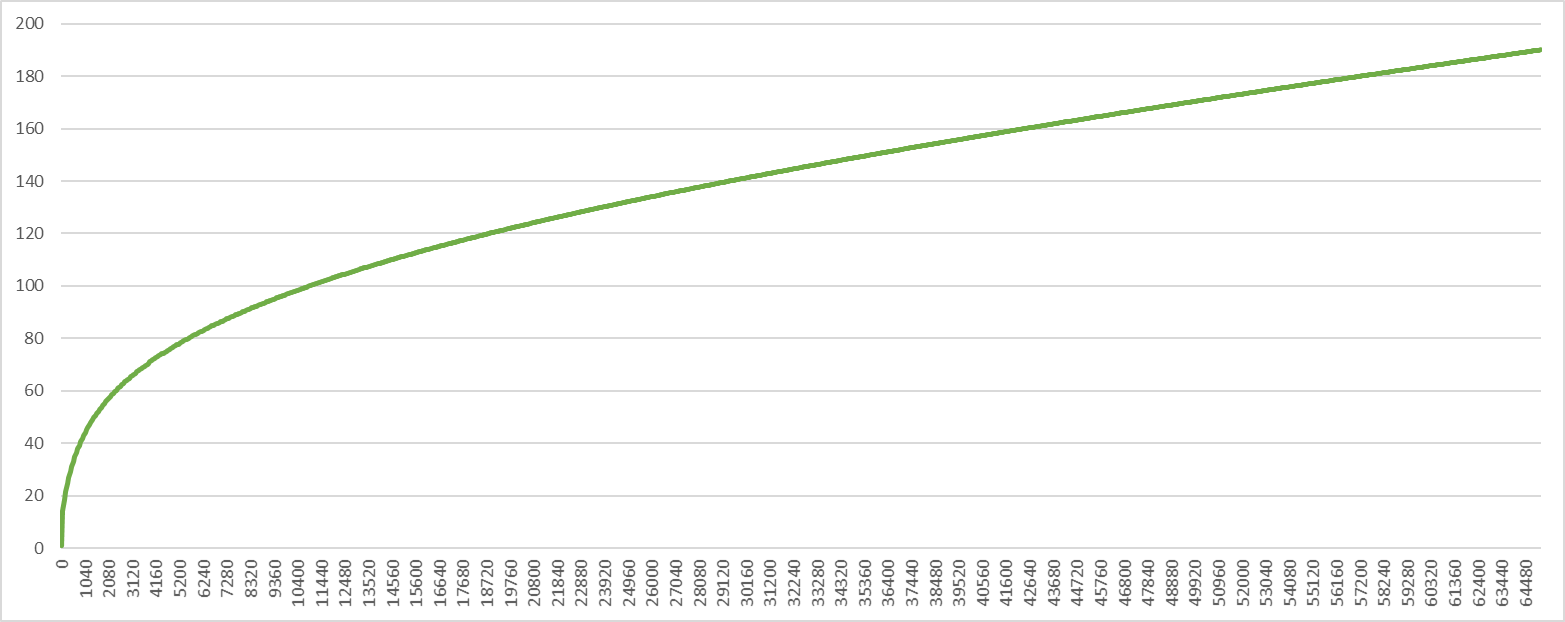
S1 Figure. Rarefaction curve of annotated species richness in visceral extract of *Haliotis discus hannai*.

α-Diversity = 9.812 species
