# Supplementary material for: Microbial community and functions associated with digestion of algal polysaccharides in the visceral tract of Haliotis discus hannai: Insights from metagenome and metatranscriptome analysis
Source: PLoS One. 2018 Oct 11;13(10):e0205594. doi: 10.1371/journal.pone.0205594 (PMC6181387; doi:10.1371/journal.pone.0205594)
Supplement: S1 Table — (DOCX) [file pone.0205594.s002.docx]

S1 Table. Summary statistics of generated whole genome sequencing data for 3 pacific abalone species.

| **Library Name** | **Library Type** | **Insert Size** | **Platform** | **Read Length** | **No. Read** | **Total bp** |
| --- | --- | --- | --- | --- | --- | --- |
| 16s amplicon | Paired-end | 550 | Miseq | 301 | 526,742 | 15,8549,342 |
| *Total Transcriptome* | Paired-end | 350 | Nextseq500 | 76 | 214,087,970 | 16,270,685,720 |
