# Supplementary material for: Microbial community and functions associated with digestion of algal polysaccharides in the visceral tract of Haliotis discus hannai: Insights from metagenome and metatranscriptome analysis
Source: PLoS One. 2018 Oct 11;13(10):e0205594. doi: 10.1371/journal.pone.0205594 (PMC6181387; doi:10.1371/journal.pone.0205594)
Supplement: S2 Table — (DOCX) [file pone.0205594.s003.docx]

S2 Table. Summary statistics for total transcriptome assembly of visceral extract using Trinity.

| **Total trinity 'genes':** | 343,970 |
| --- | --- |
| **Total trinity transcripts:** | 419,032 |
| **Percent GC** | 41.19 |
| **Stats based on ALL transcript contigs** |  |
| Contig N50 | 1,156 |
| Median contig length | 364 |
| Average contig | 703.14 |
| Total assembled bases | 294,637,012 |
| **Stats based on ONLY LONGEST ISOFORM per GENE** |  |
| Contig N50 | 714 |
| Median contig length | 339 |
| Average contig | 566.1 |
| Total assembled bases | 194,720,276 |
