# Supplementary material for: Microbial community and functions associated with digestion of algal polysaccharides in the visceral tract of Haliotis discus hannai: Insights from metagenome and metatranscriptome analysis
Source: PLoS One. 2018 Oct 11;13(10):e0205594. doi: 10.1371/journal.pone.0205594 (PMC6181387; doi:10.1371/journal.pone.0205594)
Supplement: S3 Table — (DOCX) [file pone.0205594.s004.docx]

S3 Table. Summary statistics for 16s rRNA library data used in community analysis of visceral extract of *Halitotis discus hannai*.

| **Input Sequence** |  |
| --- | --- |
| bp Count | 29,383,116 bp |
| Sequences Count | 65,139 |
| Mean Sequence Length | 451 ± 14 bp |
| Mean GC percent | 49 ± 1 % |
| **Post Quality Control** |  |
| bp Count | 29,172,480 bp |
| Sequences Count | 64,779 |
| Mean Sequence Length | 450 ± 11 bp |
| Mean GC percent | 49 ± 1 % |
| Predicted rRNA Features | 424 |
| Identified rRNA Features | 404 |
